# Differential transmission of Asian and African Zika virus lineages by *Aedes aegypti* from New Caledonia

**DOI:** 10.1038/s41426-018-0166-2

**Published:** 2018-09-26

**Authors:** Elodie Calvez, Olivia O’Connor, Morgane Pol, Dominique Rousset, Oumar Faye, Vincent Richard, Arnaud Tarantola, Myrielle Dupont-Rouzeyrol

**Affiliations:** 1 Institut Pasteur de Nouvelle-Calédonie, URE-Dengue et autres Arboviroses, Réseau International des Institut Pasteur, Nouméa, New Caledonia; 2 Institut Pasteur de Nouvelle-Calédonie, URE-Entomologie Médicale, Institut Pasteur de Nouvelle-Calédonie, Réseau International des Institut Pasteur, Noumea, New Caledonia; 3 Institut Pasteur de la Guyane, Laboratoire de Virologie, Centre National de Référence des arbovirus, Réseau International des Institut Pasteur, Cayenne, French Guiana; 4 Institut Pasteur de Dakar, Unité Arbovirus et Virus des Fièvres Hémorragiques, Réseau International des Institut Pasteur, Dakar, Senegal; 5 Institut Pasteur de Nouvelle-Calédonie, Direction, Réseau International des Institut Pasteur, Nouméa, New Caledonia; 6 Institut Pasteur de Nouvelle-Calédonie, Unité Epidémiologie, Réseau International des Institut Pasteur, Nouméa, New Caledonia

## Abstract

Zika virus (ZIKV) is a *Flavivirus* that is transmitted to humans by *Aedes* mosquitoes. ZIKV is divided into two phylogenetic lineages, African and Asian. In the Asian lineage, Pacific and American clades have been linked to the recent worldwide outbreak of ZIKV. The aim of this study was to measure the vector competence of *Aedes aegypti* for seven ZIKV strains belonging to both lineages. We demonstrate that *Ae. aegypti* from New Caledonia (NC), South Pacific region, is a low-competence vector for Asian ZIKV (<10% transmission efficiency). No significant differences were observed in vector competence with respect to the sampling date and collection site of Asian ZIKV strains used (2014 and 2015 for New Caledonia, Pacific clade, and 2016 for French Guiana, American clade). The ability of the New Caledonian *Ae. aegypti* to transmit ZIKV is significantly greater for the earlier viral isolates belonging to the African lineage (>37% transmission efficiency after 9 days post-infection) compared to recent ZIKV isolates from African (10% transmission efficiency) and Asian lineages (<10% transmission efficiency). The results of this study demonstrate that *Ae. aegypti* from NC can become infected and replicate different ZIKV strains belonging to all lineages. Our data emphasize the importance of studying the interaction between vectors and their arboviruses according to each local geographic context. This approach will improve our understanding of arbovirus transmission to prevent their emergence and improve health surveillance.

## Introduction

Zika virus (ZIKV; family *Flaviviridae*, genus *Flavivirus*) was first isolated in the eponymous Ugandan forest in 1947^1^. Since first being isolated, ZIKV has been sporadically detected in Africa and Asia and has recently emerged in the Pacific region and spread to Latin America^2^, causing major outbreaks. ZIKV is transmitted to humans by the bite of mosquitoes belonging to the *Aedes* genus, such as *Aedes aegypti* and *Aedes albopictus*^3–6^. Zika fever is a predominantly mosquito-borne disease, although rarer forms of ZIKV transmission have been described, including sexual or blood-borne transmission and vertical transmission from mother to child^7^. During the recent emergence, ZIKV infection has been associated with a dramatic increase in congenital abnormalities, including microcephaly and neurological disorders, especially in Brazil and in French Polynesia^8–11^. Previous phylogenic studies identified two distinct lineages for ZIKV, African, and Asian, which circulated in these two geographic contexts. However, the Asian lineage is solely responsible for the current global expansion of ZIKV^12–16^. Two clades have been identified in the Asian lineage, Pacific and American^14,15^, which exhibit a low genetic divergence of less than 12% at the nucleotide level^2^.

The recent explosive ZIKV epidemic in the Pacific and in South America is driven by complex and poorly understood interactions between the human host, the mosquito vector and ZIKV. Among the possible factors involved in ZIKV emergence, the characteristics of the virus, especially genetic factors and affecting vector transmission, may affect the spread of the arbovirus by impacting mosquitoes’ vector competence^17–19^. The vector competence of *Ae. aegypti* for dengue virus (DENV) is likely governed to a large extent by mosquito-virus genetic interactions, where the outcome of vector infection depends on the specific mosquito-virus genotype combination^20^. The *Ae. aegypti* genus has been shown to be composed of several subpopulations that display specific genetic characteristics^21^, even at the Pacific regional scale^22^. These characteristics, along with the genetic specificity of viruses, can impact vector competence^20,23,24^. This phenomenon has also been demonstrated for other arboviruses, such as chikungunya virus (CHIKV)^17,19,25^ and West Nile virus^26^. Recent studies have evaluated the vector competence of different vectors for several ZIKV strains isolated in Africa, Asia, Pacific, and the Americas^27^. These studies have yielded varying results depending on the vector being tested (*Ae. aegypti* or *Ae. albopictus*) and the ZIKV lineage used, emphasizing the importance of the above-mentioned mosquito-virus interactions.

New Caledonia (NC) is a French territory in the western part of the South Pacific with a population of 270,000. A Zika outbreak occurred in NC in 2014–2015 with more than 1500 confirmed cases^28,29^. Since the first reported dengue epidemic (1883), *Ae. aegypti* has been considered to be the primary vector of DENV, ZIKV and CHIKV in NC^30,31^. Previous vector competence studies quantified the ability of *Ae. aegypti* from NC to transmit DENV^32^ and  CHIKV^25^.

The aim of this study was to evaluate the potential of different ZIKV strains to infect, disseminate and be transmitted by field-collected *Ae. aegypti* from NC to expand our knowledge of mosquito-ZIKV interactions. For this purpose, four Asian lineage ZIKV strains belonging to the Pacific clade (New Caledonia) or the American clade (French Guyana) and three strains belonging to the African lineage (one old strain from Uganda and old and recent strains from Senegal) were used. We performed experimental infections using these seven ZIKV strains to document and quantify the ability of New Caledonian *Ae. aegypti* to transmit Pacific, American, and African ZIKV strains and to measure the impact of ZIKV lineage and genetic divergence on *Ae. aegypti* vector competence.

## Results

### Low competence of the New Caledonian *Aedes aegypti* mosquito for ZIKV that circulated during the New Caledonia outbreak

**Fig. 1:**
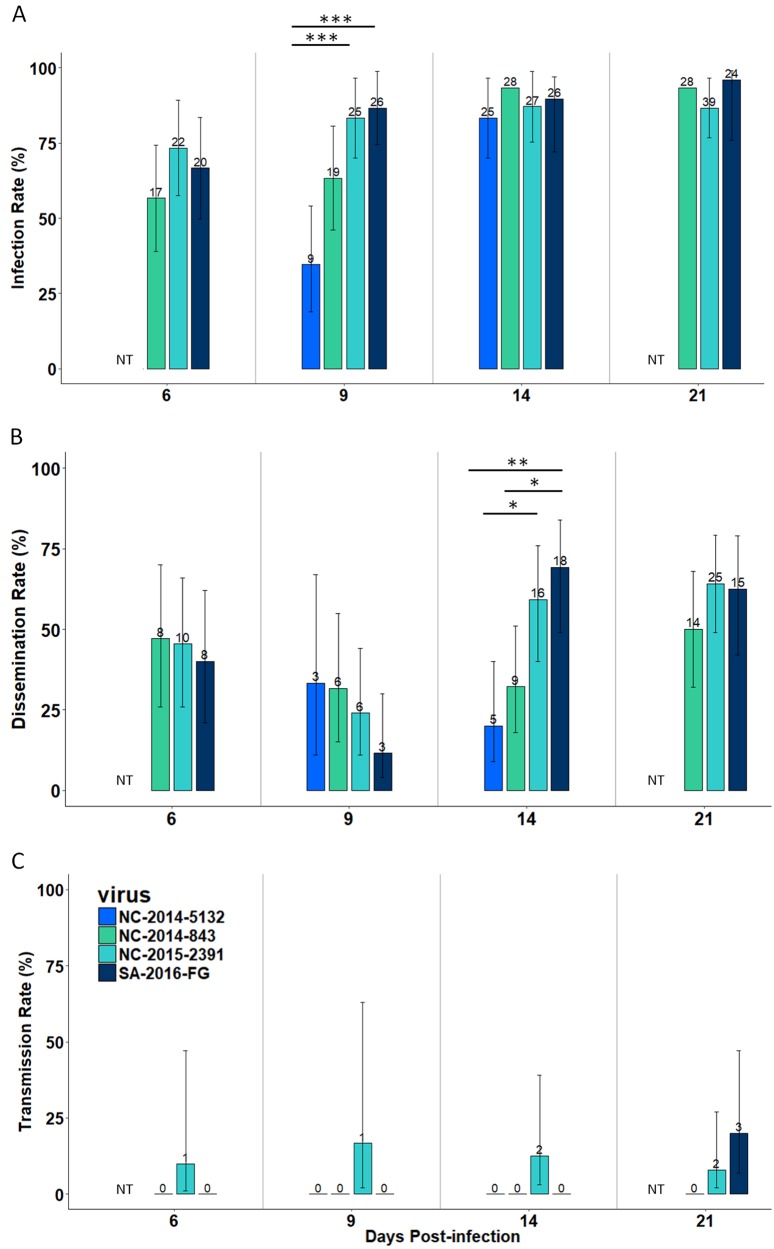
Infection, dissemination, and transmission of *Aedes aegypti* from New Caledonia with ZIKV belonging to the Asian lineage (Pacific and American clades). Infection rate (**a**), dissemination rate (**b**) and transmission rate (**c**) at 6, 9, 14, and 21 days post-infection. The number of positive mosquitoes are indicated above each bar plot. Significant differences are indicated by asterisks (*p < 0.05; **p < 0.01; and ***p < 0.001). The bars indicate the 95% confidence interval for each ZIKV strain. NT indicates that females were not tested for this analysis point

To first evaluate the vector competence of the NC *Ae. aegypti* for the Pacific ZIKV clade, we infected female mosquitoes with three ZIKV strains isolated in NC in 2014 (NC-2014-843 and NC-2014-5132) and 2015 (NC-2015-2391) (Table 1). The proportion of mosquitoes infected was high and homogenous after 14 days post-infection (dpi), 83% for NC-2014-5132 and 93% NC-2014-843 (Fig. 1a; Table S1). The dissemination rate was moderate (<69%) for the three ZIKV strains from New Caledonia (Fig. 1b; Table S1). Infection at 9 dpi and the dissemination rates at 14 dpi were significantly higher for strain NC-2015-2391 than strain NC-2014-5132 (Fisher’s exact test, *p* < 0.001 and *p* = 0.02, respectively). Infectious viral particles were only present in mosquito saliva for strain NC-2015-2391 and were detected as early as 6 dpi (Fig. 1c; Table S1). Transmission efficiencies were below 10% at each dpi tested (Fig. 3a; Table S2).

**Table 1:** Zika virus strains used in this study

ZIKV strain	Country	Year of collection	Host origin	Lineage/Clade	Passage history	GenBank Accession number	References
NC-2014-843	New Caledonia	February 2014	Human	Asian/Pacific	Three passages^(a)^	SRR5309456	Dupont-Rouzeyrol et al.^14^
NC-2014-5132	New Caledonia	April 2014	Human	Asian/Pacific	Four passages^(a)^	SRR5309451	Dupont-Rouzeyrol et al.^14^
NC-2015-2391	New Caledonia	July 2015	Human	Asian/Pacific	Four passages^(a)^	SRR5309449	Dupont-Rouzeyrol et al.^14^
SA-2016-18246	French Guiana	January 2016	Human	Asian/American	Twelve passages^(c, a)^	KU758873	Enfissi et al. GenBank direct submission
AF-1947-MR766	Uganda	April 1947	Monkey	African	Unknown^(b, c, a)^	KY989511	Faye et al.^12^
AF-1991-HD78788	Senegal	February 1991	Human	African	Unknown^(b, c, a)^	KF383039	Faye et al.^12^
AF-2002-ArD 165 522	Senegal	October 2002	*Aedes vittatus*	African	Eight passages^(b, c, a)^	KF383029	Faye et al.^12^

Passage were conducted with Vero E6 cells^(a)^, AP-61^(b)^ cells, or C636 cells^(c)^. All viral stocks used for vector competence studies were ultimately produced using Vero E6 cells

### Similar transmission efficiencies for the latest Pacific and American ZIKV strains

To further evaluate the ability of NC *Ae. aegypti* to transmit ZIKV belonging to the Asian lineage, we infected NC mosquitoes with a ZIKV strain isolated in French Guiana (SA-2016-18246) in 2016 that belongs to the American ZIKV expansion (Table 1). The observed *Ae. aegypti* infection and dissemination rates between Pacific and American strains were equivalent (Fig. 1a, b; Table S1). At 9 dpi, the infection rate was higher for the SA-2016-18246 strain than for strain NC-2014-5132 (Fisher’s exact test, *p* < 0.001) (Fig. 1a; Table S1), and at 14 dpi, dissemination rate was higher for SA-2016-18246 than for NC-2014-843 (Fisher’s exact test, *p* = 0.014) or NC-2014-5132 (Fisher’s exact test, *p* = 0.001) (Fig. 1b; Table S1). Infectious viral particles were only detected in the saliva at 21 dpi for SA-2016-18246 (Fig. 1c; Table S1). Similar to the Pacific clade, the transmission efficiency of the American clade was low (12%) (Fig. 3a; Table S2). No significant differences in transmission efficiencies were observed between the American and Pacific ZIKV clades, although infectious particles were detected in the saliva from 6 dpi for the Pacific clade (NC-2015-2391) compared to 21 dpi for the American clade (SA-2016-18246) (Fig. 3a; Table S2).

### Vector competence for the African lineage is strain-specific

**Fig. 2:**
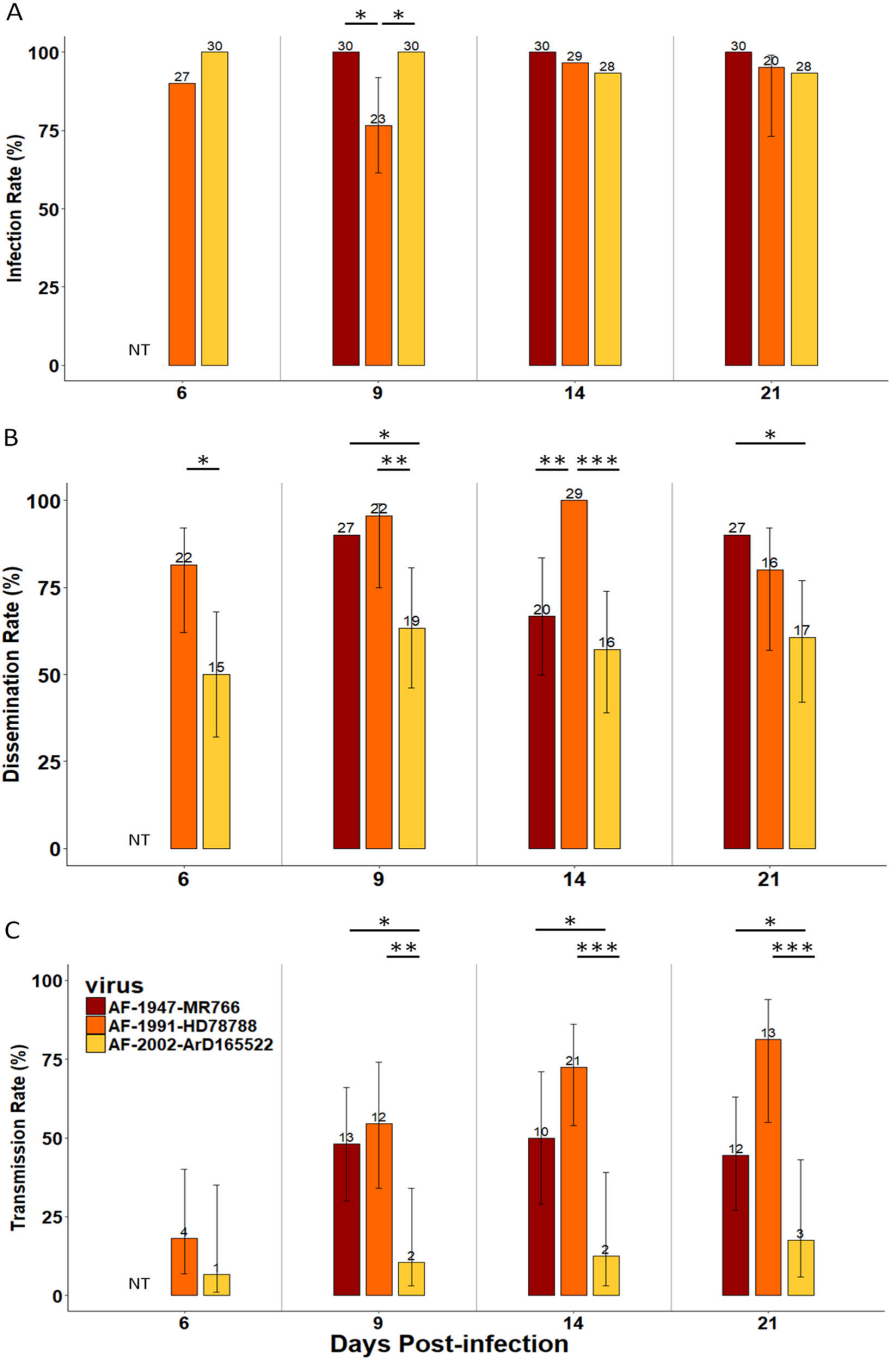
Infection, dissemination and transmission of *Aedes aegypti* from New Caledonia with ZIKV belonging to the African lineage Infection rate (**a**), dissemination rate (**b**) and transmission rate (**c**) at 6, 9, 14, and 21 days post-infection. The number of positive mosquitoes are indicated above each bar plot. Significant differences are indicated by asterisks (*p < 0.05, **p < 0.01, and ***p < 0.001). The bars indicate the 95% confidence interval for each ZIKV strain. NT indicates that females were not tested for this analysis point

**Fig. 3:**
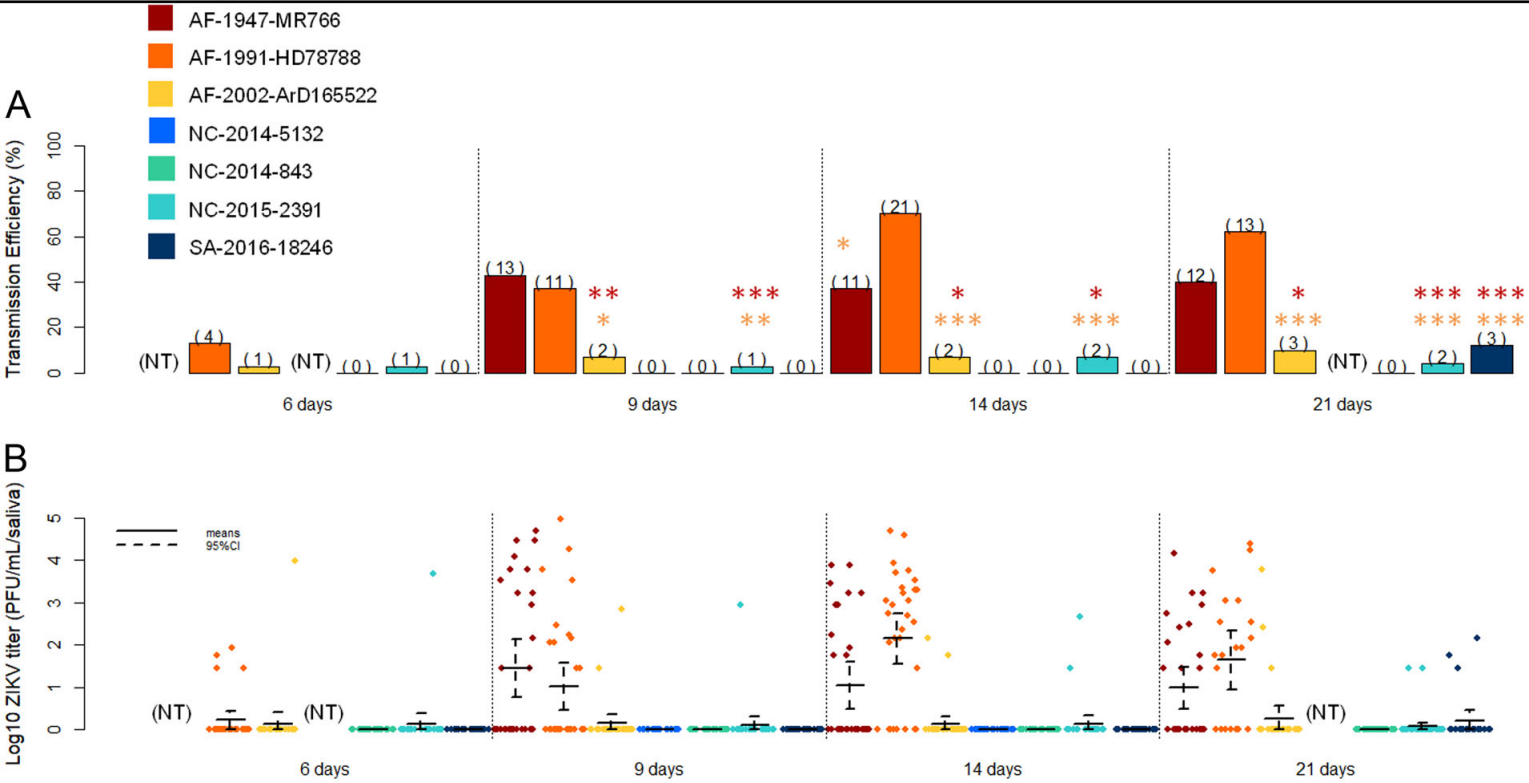
Comparison of ZIKV strain transmission by *Aedes aegypti* from New Caledonia. **a** Transmission efficiency of ZIKV by NC *Ae. aegypti* at 6, 9, 14, and 21 days post-infection. The number of positive mosquitoes are indicated above each bar plot. Significant differences are indicated by asterisks (*p < 0.05, **p < 0.01, and ***p < 0.001), in red for differences with the strain AF-1947-MR766 and in orange for the differences with the strain AF-1991-HD78788. **b** Saliva viral loads of each ZIKV strain at 6, 9, 14, and 21 days post-infection. The bars indicate the confidence interval of the mean for each ZIKV strain

In parallel, we determined the ability of NC *Ae. aegypti* to transmit ZIKV belonging to the African lineage. To this end, we performed infections with three ZIKV strains isolated in Uganda (an old strain, AF-1947-MR766, isolated in 1947) and in Senegal (an old strain, AF-1991-HD78788, isolated in 1991, and a recent strain, AF-2002-ArD 165 522, isolated in 2002) (Table 1). The proportion of infected mosquitoes was high and homogenous (between 77 and 100% of infection) over the 21 days of the analysis (Fig. 2a; Table S1). ZIKV dissemination throughout the mosquito body was moderate to high (50–100% dissemination) (Fig. 2b; Table S1). Among the three strains tested, ZIKV AF-2002-ArD 165 522 (isolated in Senegal in 2002) exhibited lower dissemination rates than the other two strains, with significant differences observed at each dpi. Transmission efficiencies for the strains AF-1991-HD78788 (isolated in Senegal in 1991) and AF-1947-MR766 (isolated in Uganda in 1947) were high (Fig. 3a; Table S2). Indeed, for the ZIKV strains AF-1947-MR766 and AF-1991-HD78788, ZIKV transmission efficiencies ranged between 37 and 72% at 9 dpi, and significant differences were observed compared to the AF-2002-ArD 165 522 strain, for which transmission efficiencies of lower than 10% in the assayed NC *Ae. aegypti* (Fig. 3a; Table S2).

### Recent ZIKV strains are less easily transmitted than older ZIKV strains

When analyzing all the experimental infections, our results showed that NC *Ae. aegypti* more easily transmit older ZIKV strains belonging to the African lineage (AF-1947-MR766 and AF-1991-HD78788) than the other strains tested (Fig. 3a: Table S1–S2). Initially, significant differences were observed in the infection and dissemination rates between ZIKV belonging to the African (AF-1947-MR766, AF-1991-HD78788 and AF-2002-ArD165 522) and that of the Asian lineages (NC-2014-843, NC-2014-5132, NC-2015-2391, and SA-2016-18246). Infection rates before 14 dpi were significantly higher for the African ZIKV compared to the strains of the Pacific and American clades (Fig. 1a, 2a; Table S1). The three African ZIKV strains appeared to disseminate more than the Asian lineage strains in the New Caledonian *Ae. aegypti*, especially at 9 and 14 dpi (Fig. 1b, 2b; Table S1). However, significant differences in transmission efficiency were observed between the recent ZIKV strains (Asian lineage and AF-2002-ArD 165 522) and the two older ZIKV strains of the African lineage (AF-1947-MR766 and AF-1991-HD78788) from 9 dpi (Fisher’s exact test, *p* < 0.05) (Fig. 3a; Table S2).

### High transmission efficiency is correlated with high ZIKV titer in *Aedes aegypti* saliva

We quantified the number of infectious viral particles in saliva for the five ZIKV strains that were transmitted by NC *Ae. aegypti*. Higher ZIKV titers were observed in the saliva of mosquitoes infected with the older African lineage strains AF-1947-MR766 and AF-1991-HD78788 (Fig. 3b), and significant differences were observed between these two strains and the three other strains (AF-2002-ArD 165 522, NC-2015-2391 and SA-2016-18246) after 9 dpi (Kruskal–Wallis test, *p* < 0.05, degrees of freedom = 1).

## Discussion

A mosquito is considered to be a vector if it is able to transmit a pathogen. Vector competence is determined by the ability of a virus to replicate and disseminate through the body of a mosquito before reaching its salivary glands, thus enabling transmission upon subsequent blood feeding^33^. For this purpose, the virus needs to infect and escape both the midgut and salivary gland barriers. Our study shows that ZIKV particles were present in mosquito saliva as early as 6 dpi and that NC *Ae. aegypti* is a competent ZIKV vector. We also demonstrated that NC *Ae. aegypti* vector competence for ZIKV greatly varies according to the ZIKV strain considered.

In our study, infection and dissemination rates were higher for ZIKV belonging to African lineage compared to the Asian lineage (Pacific and American clades). Interestingly, mosquitoes displaying a higher transmission efficiency also had higher viral titers in their saliva (Fig. 3b). The observed differences in infection and dissemination suggest the influence of ZIKV strain-specific differences in midgut barrier escape. These differences may also be linked to ZIKV-specific viral replication limitations in the body and secondary organs of the mosquito^34^. The number of infected cells in the mosquito midgut epithelium can vary depending on the virus-mosquito combination^34^. For example, less than 30% of the *Culex quinquefasciatus* midgut cells are infected with West-Nile virus^35^, and the wall midgut of *Culex tritaeniorhynchus* appeared to be infected by Japanese Encephalitis virus^36^.

We observed a low transmission rate for the recent ZIKV strains belonging to the African and Asian lineages, indicating that the salivary gland barrier has a role in ZIKV transmission by this population of *Ae. aegypti*^34^. Although the infectious blood-meal provided was at a high titer (10^7^ TCID_50_/mL), this salivary gland barrier may have influenced the documented transmission rate. Previous studies highlighted the impact of viral fitness and transmission of the salivary gland and the bottleneck effect of the midgut barrier on viral particles during the transmission process^37^. Allowing specific selection of viral subspecies can decrease the number of viral particles or reduce the effective virus population size. This effect was previously described containing a Venezuelan Equine Encephalitis virus clone, where a significant drop in the number of clones was observed at each step of the mosquito infection^38,39^. The lower infection and dissemination rates observed for *Ae. aegypti* from NC infected by recent ZIKV strains (African: AF-2002-ArD 165 522; Asian: NC-2014-843, NC-2014-5132, NC-2015-2391, and SA-2016-18246) compared to older African ZIKV strains (AF-1947-MR766 and AF-1991-HD78788) can be explained by efficient barriers and/or the innate immune system of *Ae. aegypti* modulating or limiting ZIKV transmission. Previous studies have described the influence of the mosquito immune system on arbovirus transmission^40,41^. Moreover, specific interactions between the immune system and viral strains was observed for dengue virus in a previous study^42^. ZIKV transmission by *Aedes* mosquitoes was experimentally estimated using several vector populations from around world (Europe, Asia, America, and Pacific) and the Asian ZIKV lineage^3,27,43,44^. Overall, these studies showed low transmission efficiencies, as was observed in our study^27^. The ZIKV isolated during the NC 2013-2016 outbreak appears to be able to infect and replicate in the bodies of vectors, although the salivary glands, appear to be a major barrier to transmission in NC *Ae. aegypti* mosquitoes.

Phylogenetic analyses comparing the Pacific and American ZIKV clades have identified amino acid substitutions, although genetic divergence is low. Only one amino acid substitution on the NS5 genome region has been identified^14,15^. However, in our study, these viral genetic differences apparently did not impact *Ae. aegypti* vector competence. We observed no increase in transmission between ZIKV strains isolated during the initial (in New Caledonia: NC-2014-843, NC-2014-5132 and NC-2015-2391) or later (French Guiana: SA-2016) recent ZIKV outbreaks. Furthermore, the level of observed ZIKV transmission remained low, with no significant differences observed, contrary to a recent study by Pompon et al^45^. In that study, significant differences were observed in transmission efficiency of Asian *Ae. aegypti* for a ZIKV strains from Brazil and French Polynesia. However, in that study^45^, molecular techniques were used for transmission analysis, which might have increased the sensitivity of the results. Although less sensitive, our plaque assay allowed us to observe the infectivity of the saliva. The higher transmission rate of the Brazilian strain was associated with a higher viral titer in the vector body and in the saliva at 10 dpi. This finding is in agreement with those of previous studies and shows the impact of the viral titer on ZIKV escape of the midgut barrier^34,46^. The role of mutations on the infection/transmission ability of ZIKV needs to be assessed.

In addition, the transmission of the AF-1947-MR766 strain was described in several previous studies (using the same viral titer as our study), the result of which indicated moderate dissemination with *Aedes hensilli* from Yap island^6^, high transmission with *Aedes* vectors from Mexico^47^ and Singapore^48,49^, and no transmission with *Ae. aegypti* from Senegal^4^. These results, as well as those obtained in our study, highlight the specific virus-vector interactions observed for this ZIKV strain. For strain NC-2014-5132 (belonging to the Asian lineage and the Pacific clade), the results are more coherent using *Aedes* vectors, as low transmission was observed for *Ae. aegypti* and *Ae. albopictus* from Western Europe^43^ and Brazil^3^, as was shown by our own results.

Furthermore, the importance of this mosquito-virus interaction was underscored by the higher ZIKV transmission observed for *Ae. aegypti* from NC with the old ZIKV strains belonging to the African lineage (AF-1947-MR766 and AF-1991-HD78788). These results are in accordance with other studies, most of which observed higher transmission by *Aedes* mosquitoes of ZIKV belonging to the African lineage^47,50^, except with the *Aedes* mosquitoes from Senegal, where low dissemination and no transmission was reported for the two *Ae. aegypti* populations tested^4^. In the latter study, the same ZIKV strains (AF-1947-MR766, AF-1991-HD78788 and AF-2002-ArD 165,522) were used with the same blood meal titer as in our study to assess the ZIKV vector competence of several F1 generation *Aedes* species from Senegal. Both of these studies emphasize that for a given virus strain, different geographic origins lead to genetic differentiation within the same vector species (here *Ae. aegypti*) that may impact virus transmission^51^. Finally, in our study, the transmission efficiency of one ZIKV strain of the African lineage (AF-2002-ArD 165,522) was lower than the two assayed. This strain was isolated from *Aedes vittatus* in 2002 in Senegal.

Our study may suffer from bias and limitations. The vector competence results we obtained may have been determined by interactions between the specific virus and vector genotypes, as ZIKV AF-2002-ArD 165,522 is the only strain isolated from an arthropod (as opposed to mammals for all others) and was the only recent African lineage strain assayed. Although genetic variations could explain the results obtained using the different ZIKV strains in our study, these could have been affected by other factors, such as viral production and passage history. Parameters such as passage number and cell type could have modulated virus populations and then reduced or increased viral fitness in the vector during in vivo experiments^52,53^. The genetic divergence and higher number of passages could explain the transmission differences between strains AF-1947-MR766 and AF-1991-HD78788 and the other strains, especially the ability of these two strains to infect and escape the salivary gland barrier. Whether and how these parameters may have influenced the experimental infection results should be investigated. Such an investigation could be conducted by constructing mutagenized ZIKV isolates and measuring their impact on infection or vector competence, as has been previously described for other arboviruses^54,55^.

The results of our study show that *Ae. aegypti* from NC is a competent but not a highly efficient vector of recent ZIKV strains. An epidemic nevertheless occurred in NC due to the high density of vectors, their lifespan, and close contacts between humans and *Aedes* mosquitoes. Furthermore, the emergence of Asian ZIKV lineages in the Pacific and in the Americas may be linked to other factors, such as changing patterns of human migration and behavior, large immunologically naive fractions of the population and urbanization rather than vector competence alone. This ZIKV emergence draws a parallel with the 2014 CHIKV outbreaks in the Pacific region and in the Americas caused by the Asian lineage of CHIKV^56,57^. The geographical expansion of arbovirus vectors and the intensification of international exchanges contribute to the dissemination of arboviruses in subtropical and temperate areas^58^. The introduction and circulation of DENV, ZIKV, or other *Flaviviruses* such as Yellow Fever^59^ virus is a major concern. Our results underscore the importance of studying the specific interactions between field vectors and arboviruses in each specific geographical context to anticipate public health risks.

## Material and methods

### Mosquito collection

Mosquitoes were sampled at the immature stage in Noumea from an artificial breeding site during the hot season. Approximately 200 larvae identified as belonging to *Ae. aegypti* species were placed in a 2 L plastic container and feed with brewer’s yeast capsules. Pupae were secondly transferred to a cage (30-cm sides) to await adult emergence. Females were blood-fed twice a week for at least five weeks with guinea pig blood to obtain F1-generation eggs. The F2-generation eggs were obtained under similar conditions (approximately 150 larvae per plastic container and 400 to 600 mosquitoes per cage). The room was maintained at 28 °C and 80% humidity under a 12:12 h light-dark cycle throughout the rearing process, with adult-stage mosquitoes fed a 10% sucrose solution ad libitum.

### Viral strain

Seven ZIKV strains were used in this study (Table 1). Three strains were isolated from patients in New Caledonia (NC) during the 2014-2015 outbreak, including two at the beginning of the outbreak in February and April 2014 (NC-2014-843 and NC-2014-5132, respectively) and one toward the end of the outbreak in July 2015 (NC-2015-2391)^14^. The ZIKV strain from the American clade was obtained from a patient blood sample in French Guyana in January 2016 (SA-2016-18246). Three strains were collected in Africa; the AF-1947-MR766 strain was isolated from a rhesus macaque in Zika Forest in Uganda in 1947;^4,12^ the AF-1991-HD78788 strain was isolated from a patient in Senegal in 1991;^4,12^ and the AF-2002-ArD 165 522 strain was collected from *Aedes vittatus* in Senegal in 2002^4,12^. All final viral stocks were prepared with passages in mammalian Vero E6 cells in DMEM medium (Gibco^™^, Fisher scientific, UK) with 10% fetal bovine serum (FBS; Gibco™, Fisher scientific) for 3–5 days at 37 °C with 5% CO_2_. Supernatants were collected and stored at −80 °C. Viral titers were determined using serial 10-fold dilutions of viral stocks on Vero E6 cells and were expressed as TCID50/mL for each viral stock.

### Mosquito oral infections

Five-to seven days-old females (not previously blood-fed) were starved for 24 h before infection. The mosquitoes were allowed to take an infectious blood meal for 20 min using an artificial system with pig intestine membrane containing 2 mL of washed rabbit erythrocytes and 1 mL of viral suspension supplemented with 5 mM adenosine triphosphate (Sigma-Aldrich^®^, Germany) as a phagostimulant. To optimize and compare the impact of ZIKV lineages on vector competence, the final concentration of ZIKV used in the blood meal was 10^7^ TCID50/mL, as previously described^3,43,44^. After taking the blood meal, fully engorged females were transferred into new containers and maintained at 28 °C and 80% humidity, with a 12:12 h light-dark cycle and ad libitum access to 10% sucrose.

### Infection, dissemination, and transmission analysis

For each viral strain tested, 20–30 females were analyzed at 6, 9, 14, 21 days post-infection (dpi). The females were cold-anesthetized and had their legs and wings removed and discarded. For salivation, the proboscis of each female was inserted into a filter tip containing 5 µL of FBS for 20 min, and samples were subsequently stored at −80 °C. After salivation, the head was cut from the body (abdomen and thorax). For each mosquito, the body, head, and saliva were used to determine the infection, dissemination, and transmission rates. Each body and head were individually and mechanically ground with 2.4 mm metal beads in 250 µL of DMEM medium supplemented with 2% FBS. The materials were ground for 30 s at 6000 rpm using a MagnaLyser (Roche Diagnostics^®^, Switzerland). The samples were centrifuged at 10,000×*g* for 10 min at 4 °C. The supernatants were collected and stored at −80 °C before analysis. For viral detection, 100 µL of the head and body samples were inoculated onto Vero E6 cells in 96-well plates, incubated at 37 °C with 5% CO_2_ for 7 days, and were subsequently stained with a 0.2% crystal violet solution (in 10% formaldehyde and 20% ethanol). Viral particles were detected by the presence of a cytopathic effect (CPE). Saliva samples were inoculated directly after salivation onto Vero E6 cells in 6-wells plates under a 2% agarose overlay and were incubated at 37 °C with 5% CO_2_ for 7 days, after which samples were processed as previously described. Saliva titers were expressed as plaque forming units (pfu) per milliliter of saliva.

### Ethical clearance

The experiments performed in this study observed the New Caledonia ethic regulations regarding animal experiments. NC virus isolates used in this study were previously obtained from the sera of anonymized patients who were unopposed to the secondary use of their biological material. The ZIKV isolate from French Guiana was obtained as part of surveillance and expertise activities of the Arboviruses National Reference Center in French Guiana.

### Statistical analysis

The infection rate (number of positive bodies divided by the total number of mosquitoes tested), dissemination rate (number of infected heads divided by the number of infected bodies), transmission rate (number of infected saliva samples divided by the number of infected heads) and transmission efficiency (number of infected saliva samples divided by the total number of mosquitoes tested) were calculated for each ZIKV strains at each dpi.

The samples were considered positive when the presence of ZIKV particles (presence of CPE) was detected in saliva regardless of the titer being tested and negative in absence of CPE. The number of positive samples allowed the positivity rates to be calculated and compared by virus and by days post-infection. The mean and median ZIKV particle titers were also calculated and compared by viral strain and by day post-infection.

Statistical analyses were performed with R v. 3.3.1 (R Core Team, Vienna, Austria)^60^. Qualitative variables were compared using Fisher’s exact test, and quantitative variables by non-parametric tests (Kruskal–Wallis test) because of a non-normal distribution. Differences were considered significant for *p*-values ≤ 0.05.

## Electronic supplementary material

Table S1: Positivity rates of infection, dissemination and transmission at 6, 9, 14 and 21 days post-infection for the ZIKV tested in this study

Table S2: Means of ZIKV titers for transmission at 6, 9, 14 and 21 days post-infection for the different studied viruses
