# Supplementary material for: Differential transmission of Asian and African Zika virus lineages by Aedes aegypti from New Caledonia
Source: Emerg Microbes Infect. 2018 Sep 26;7:159. doi: 10.1038/s41426-018-0166-2 (PMC6156223; doi:10.1038/s41426-018-0166-2)
Supplement: Supplementary file 1 — Table S1: Positivity rates of infection, dissemination and transmission at 6, 9, 14 and 21 days post-infection for the ZIKV tested in this study [file 41426_2018_166_MOESM1_ESM.pdf]

Table S1: Positivity rates of infection, dissemination and transmission at 6, 9, 14 and 21 days post-infection for the ZIKV tested in this study.

Day post infect°		6 dpi			9 dpi			14 dpi			21dpi		
Virus	n	Positive	(%)	n	Positive	(%)	n	Positive	(%)	n	Positive	(%)	P-value
**Asian Lineage**	**Infection (Number of infected bodies / number of mosquitoes tested)**												
NC-2014-843	30	17	(57)	30	19	(63)	30	28	(93)	30	28	(93)	**<0.001**
NC-2014-5132	--	NT	--	26	9	(35)	30	25	(83)	--	NT	--	**<0.001**
NC-2015-2391	30	22	(73)	30	25	(83)	29	25	(86)	45	39	(87)	0.47
SA-2016-18246	30	20	(67)	30	26	(87)	29	26	(90)	25	24	(96)	**0.02**
**African Lineage**													
AF-1947-MR766	--	NT	--	30	30	(100)	30	30	(100)	30	30	(100)	--
AF-1991-HD78788	30	27	(90)	30	23	(77)	30	29	(97)	21	20	(95)	0.09
AF-2002-ArD 165 522	30	30	(100)	30	30	(100)	30	28	(93)	30	28	(93)	0.32
P-value		**<0.001**			**<0.001**			0.19			0.36		
**Asian Lineage**	**Dissemination (number of infected heads / number of infected bodies)**												
NC-2014-843	17	8	(47)	19	6	(32)	28	9	(32)	28	14	(50)	0.15
NC-2014-5132	--	NT	--	9	3	(33)	25	5	(20)	--	NT	--	0.71
NC-2015-2391	22	10	(45)	25	6	(24)	25	14	(56)	39	25	(64)	**0.01**
SA-2016-18246	20	8	(40)	26	3	(12)	26	18	(69)	24	15	(63)	**<0.001**
**African Lineage**													
AF-1947-MR766	--	NT	--	30	27	(90)	30	22	(73)	30	27	(90)	0.15
AF-1991-HD78788	27	22	(81)	23	22	(96)	29	29	(100)	20	16	(80)	**0.04**
AF-2002-ArD 165 522	30	15	(50)	30	19	(63)	28	16	(57)	28	17	(61)	0.79
P-value		**<0.001**			**<0.001**			**<0.001**			**0.004**		
**Asian Lineage**	**Transmission (number of infected saliva / number of infected heads)**												
NC-2014-843	8	0	(0)	6	0	(0)	9	0	(0)	14	0	(0)	--
NC-2014-5132	--	NT	--	3	0	(0)	5	0	(0)	--	NT	--	--
NC-2015-2391	10	1	(10)	6	1	(17)	14	2	(14)	25	2	(8)	0.84
SA-2016-18246	8	0	(0)	3	0	(0)	18	0	(0)	15	3	(20)	**0.01**
**African Lineage**													
AF-1947-MR766	--	NT	--	27	13	(48)	22	11	(50)	27	12	(44)	0.96
AF-1991-HD78788	22	4	(18)	22	11	(50)	29	21	(72)	16	13	(81)	**<0.001**
AF-2002-ArD 165 522	15	1	(7)	19	2	(11)	16	2	(13)	17	3	(18)	0.95
P-value		0.09			**<0.001**			**<0.001**			**<0.001**		
