# Supplementary material for: Differential transmission of Asian and African Zika virus lineages by Aedes aegypti from New Caledonia
Source: Emerg Microbes Infect. 2018 Sep 26;7:159. doi: 10.1038/s41426-018-0166-2 (PMC6156223; doi:10.1038/s41426-018-0166-2)
Supplement: Supplementary file 2 — Table S2: Means of ZIKV titers for transmission at 6, 9, 14 and 21 days post-infection for the different studied viruses [file 41426_2018_166_MOESM2_ESM.pdf]

Table S2: Means of ZIKV titers for transmission at 6, 9, 14 and 21 days post-infection for the different studied viruses.

Day post infect°	6 dpi			9 dpi			14 dpi			21dpi			
Virus	n	mean	95%CI	n	mean	95%CI	n	mean	95%CI	n	mean	95%CI	P-value
**Asian Lineage**	**Transmission (number of infected saliva / number of infected heads)**												
NC-2014-843	8	0.00	--	6	0.00	--	9	0.00	--	14	0.00	--	--
NC-2014-5132	--	NT	--	3	0.00	--	5	0.00	--	--	NT	--	--
NC-2015-2391	10	0.12	[0.00-0.37]	6	0.09	[0.00-0.29]	14	0.13	[0.00-0.33]	25	0.06	[0.00-0.15]	0.93
SA-2016-18246	8	0.00	--	3	0.00	--	18	0.00	--	15	0.21	[0.00-0.46]	**0.01**
**African Lineage**													
AF-1947-MR766	--	NT	--	27	1.44	[0.76-2.12]	22	1.04	[0.49-1.59]	27	0.97	[0.48-1.47]	0.59
AF-1991-HD78788	22	0.22	[0.01-0.43]	22	1.01	[0.44-1.58]	29	2.15	[1.56-2.74]	16	1.64	[0.95-2.33]	**<0.001**
AF-2002-ArD 165 522	15	0.13	[0.00-0.40]	19	0.14	[0.00-0.35]	16	0.13	[0.00-0.31]	17	0.25	[0.00-0.56]	0.80
P-value		0.06			**<0.001**			**<0.001**			**<0.001**		
